# Difference of Morphology and Immunophenotype between Central and Peripheral Squamous Cell Carcinomas of the Lung

**DOI:** 10.1155/2013/157838

**Published:** 2013-08-29

**Authors:** Tomayoshi Hayashi, Hisao Sano, Ryoko Egashira, Kazuhiro Tabata, Tomonori Tanaka, Toshiyuki Nakayama, Yukio Kashima, Takashi Hori, Sayuri Nunomura, Junya Fukuoka

**Affiliations:** ^1^Department of Pathology, Nagasaki University Hospital, Nagasaki 852-8501, Japan; ^2^Laboratory of Pathology, Toyama University Hospital, Toyama 930-0194, Japan; ^3^Department of Radiology, Faculty of Medicine, Saga University, Saga 849-8501, Japan

## Abstract

*Background*. Recent agents, that is, pemetrexed and bevacizumab, have shown reproductive negative association between squamous histology. According to these agents' effectiveness, ruling out of the squamous histology is a significant issue for surgical pathologists. Several articles have proposed the distinction of peripheral type from central type of squamous cell carcinoma (SqCC) due to its similarity to adenocarcinoma, although little evidence to support the difference between these two types was published. In this study, we compared the clinicopathologic findings of central and peripheral pulmonary SqCCs. *Material and Methods*. 15 central and 35 peripheral types of SqCC from 2005 to 2010 were examined. Twelve morphological features were scored based on their intensity in the original H&E slides, and then, tissue microarray holding triplicated cores from 43 cases was immunohistochemically examined for cytokeratin (CK)7, CK14, TTF-1, Napsin A, p63, CK34**β**E12, CK5/6, and p53. *Result*. Most of the histological findings did not separate central and peripheral SqCCs; only the presence of emphysema, interstitial fibrosis, and entrapped pneumocytes inside the tumor showed statistic predominance in peripheral SqCC. This is the first immunophenotypic research in the central and peripheral types of SqCC.

## 1. Introduction

Lung cancer is one of the largest causes of cancer death in Japan and other countries including the USA. Squamous cell carcinoma (SqCC) occupies approximately 30% in all lung cancers [1]. It can be classified into the central type squamous cell caranoma (cSqCC) and the peripheral type squamous cell caranoma (pSqCC) according to the location of the primary site. According to textbooks, the majority of SqCCs are thought to arise centrally; however, pSqCC is changing from being minor to major by recent reports [2–4]. Referring to recent reports, pSqCC is increasing up to 50% of all lung SqCCs in some of them [3, 4].

Current therapeutic progress of lung cancer requires pathological subclassification, especially careful ruling out of SqCCs [1]. There have been a few debates whether the location of SqCC may raise any difference in biological features [3]. One report indicated that there may be a difference in growth pattern between cSqCC and pSqCC [3], while morphological and biological differences between them have not been well documented. If they are biologically different, the treatment should be considered separately which may include use of pemetrexed and bevacizumab for SqCC.

Funai et al. claimed that they observed the small cSqCC showing dysplasia or carcinoma in situ adjacent to them; however, the pSqCC rarely showed such dysplastic changes [3]. This may indicate the potential differences in carcinogenesis between cSqCC and pSqCC. 

As for the staging of SqCC, there are a few issues associated with morphologic observations. For endobronchial cSqCC especially, the mass sometimes grows largely, due to longitudinal growth along bronchial space, or expands inside the bronchial lumen by expanding the space without remarkable invasion [5]. 

At present, there has been no report that compared the differences of immunohistochemical profiles between cSqCC and pSqCC. To determine if there are any differences in the histologic or biologic patterns between the two groups, we examined the morphology, immunohistochemical features, and prognostic status.

## 2. Material and Methods

**Figure 1:**
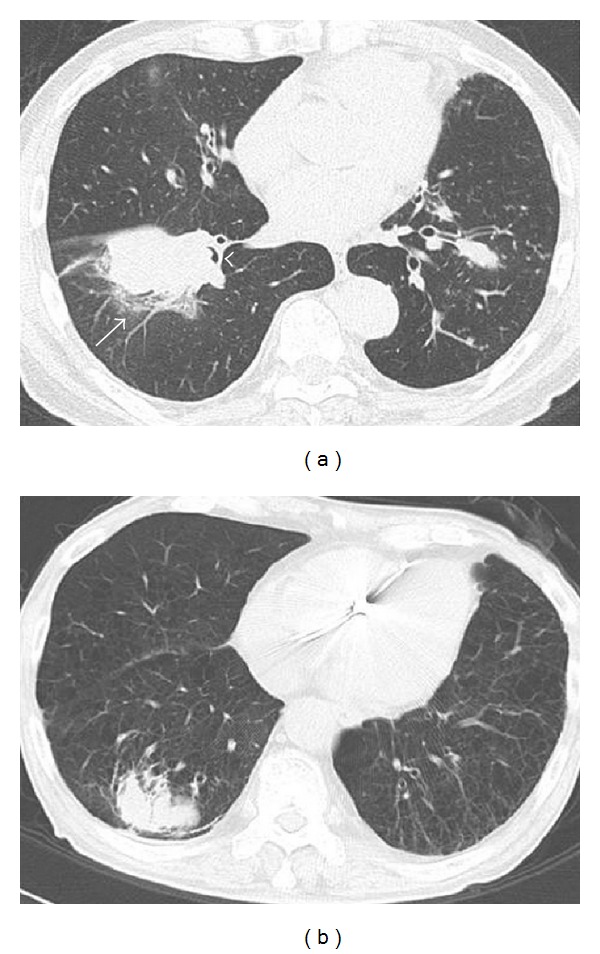
CT findings of the central and peripheral squamous cell carcinomas. (a) Central squamous cell carcinoma was defined as a lesion located from trachea to the segmental bronchi. Note bronchiolar obstruction (arrowhead) and peripheral obstructive pneumonia (arrow). (b) Peripheral squamous cell carcinoma was defined as the one located in the more peripheral location.

The cases examined were sixty lung lobectomy cases with SqCC, found in the medical files of Toyama University Hospital, at the period from 2005 to 2010, when the computed tomography (CT) data and patient's records were available through the electric medical chart system. The cases were classified into cSqCC and pSqCC by a thoracic radiologist based on the CT findings. The cSqCC was defined as a lesion located from trachea to the segmental bronchi, and the pSqCC was defined as the one located in more peripheral location (Figure 1). Among the 60 cases, 3 were excluded based on the difficulty to judge the location. Seven cases had no available CT data in the record and were also excluded. 

Pathologic reports were reviewed for age, sex, location, smoking status, pathologic stages, levels of differentiation, visceral pleural invasion, angiolymphatic invasion, and adequate margins of resection. All tumors were fixed in 10% buffered formalin, and 4 micron sections of the tumor were stained with hematoxylin and eosin (H&E) and Elastica van Gieson (EVG) staining for the observation of elastic fibers. H&E slides were reviewed to score the levels of necrosis, keratinization, inflammatory cell infiltration, alveolar filling pattern of the tumor cells, emphysema, and interstitial fibrosis outside the tumor. 

Tissue microarray containing triplicated 0.6 mm cores from each case was composed. To compensate for tissue heterogeneity, cores were taken to cover variations of histological features, such as covering range of differentiation in the same case. Immunohistochemical stains were performed with antibodies including cytokeratin 7 (CK7) (Dako, Carpinteria, CA, USA; clone, OV-TL12/30; dilution, 1 : 200), TTF-1/Napsin A (AD cocktail antibody; Pathology Institute Corp, Toyama, Japan; working solution), p63/CK14 (SqCC cocktail antibody; Pathology Institute Corp, Toyama, Japan; working solution), high molecular cytokeratin (Dako, CA, USA; 34*β*E12; 1 : 50), CK5/6 (Dako, CA, USA; D5/16 B4; 1 : 50), and p53 (Dako, CA, USA; DO-7; 1 : 50) as previously described [3]. Briefly, heat-induced antigen retrieval was applied to all antibodies at pH 9.0. Each antibody was incubated for 30 minutes at room temperature. Autostainer with polymer detection system (Envision+; Dako, CA, USA) was used for staining as manufacture recommended. Staining intensity (Intensity Score: IS) and distributions (Distribution Score: DS) inside the tumor cells were scored separately as previously described on [3]. A degree of entrapped pneumocytes inside the tumor nests of each core was also scored by observing CK7. 

The correlations between clinicopathologic characteristics and histologic subgroups were evaluated using the *χ* ^2^ test. If the number of cells was less than 5, we used the Fisher's exact test. *P* values less than 0.05 were considered significant. Data were analyzed with JMP Software (SAS, Chicago, IL; USA). Log-rank test was performed to compare the prognostic difference between cSqCC and pSqCC. Kaplan-Meier curve was plotted as well.

## 3. Results

The numbers of cSqCC (Figure 1(a)) and pSqCC (Figure 1(b)) decided by CT were 15 and 35, respectively. Strong male predominance (48 : 2) and association with smoking history (46/48) were observed (Table 1).

**Figure 2:**
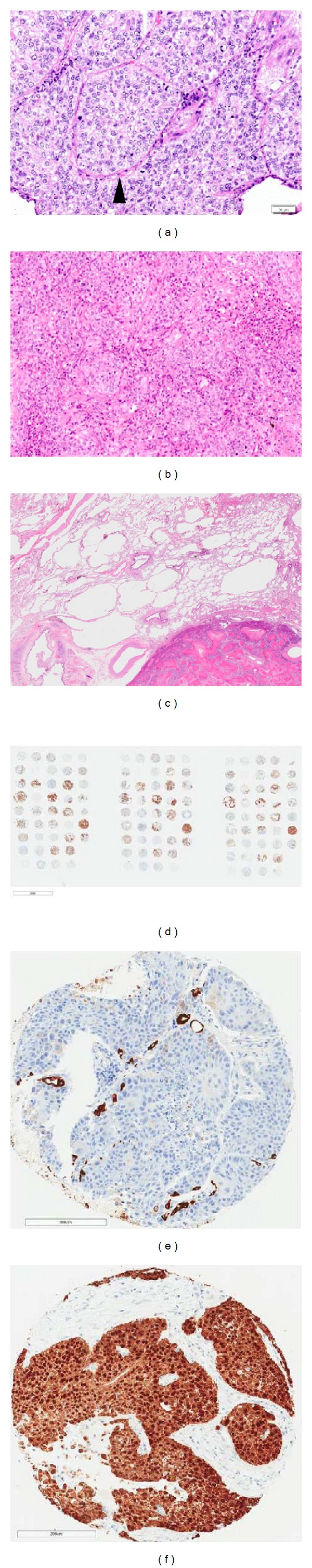
Histologic and immunohistochemical features of squamous cell carcinoma. (a) The alveolar filling pattern showing a characteristic growth of filling up the alveolar space without destruction of the alveolar network (arrowhead). (b) Histology of the infiltrating pattern. The tumor forms irregular shaped nests and is intermingled with an extensive stroma. (c) Emphysema around the tumor is seen in the majority of peripheral squamous cell carcinomas (SqCCs). (d) Scanning magnification of tissue microarray holding triplicated 43 SqCC cases (SqCC cocktail antibody staining). (e) CK7 staining shows positive for entrapped pneumocytes. (f) Most of SqCC cases were positive for p63 (nuclear) and CK14 (cytoplasmic). Diameter of all cores is 0.6 mm.

The summary of histological findings and immunohistochemical results seen in all cases was shown in Tables 2 and 3. Most of the histological findings did not separate cSqCC and pSqCC, whereas only the presence of emphysema showed statistical predominance in pSqCC (*P* = 0.02) (Table 2). Most of the histological findings such as a degree of histological differentiation and visceral pleural invasion did not separate central and peripheral SqCCs. Growth patterns such as alveolar filling pattern (Figure 2(a)) and infiltrating pattern (Figure 2(b)) were not statistically different; however, considering the low *P* value of those two findings, this lack of significance may be caused by the small sample size. Instead, abnormality in background lung such as presence of emphysema and interstitial fibrosis apart from the tumor showed statistically significant difference (Table 2). 

Out of 50 cases, paraffin blocks of 43 cases were available for tissue microarray analysis. Immunohistochemical patterns were similar between cSqCC and pSqCC (Figure 2), and most of them did not present statistically significant difference. The only significant difference was the presence of entrapped pneumocytes highlighted by CK7 which was seen mostly in pSqCC (*P* = 0.04) (Table 3). 

**Figure 3:**
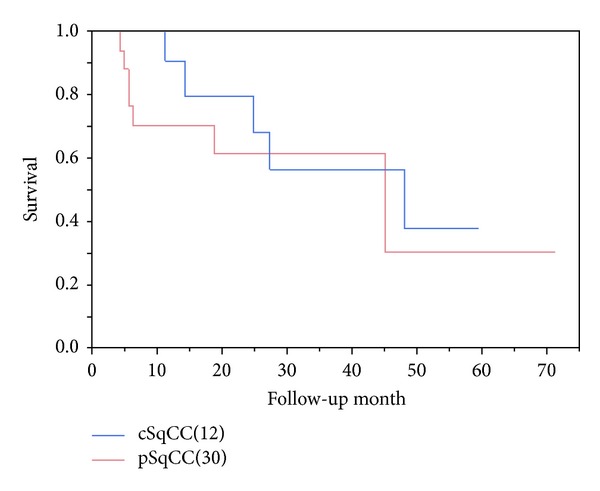
Kaplan Meier curve of central and peripheral squamous cell carcinomas. There is no significant difference in the overall survival between central and peripheral squamous cell carcinomas (*P* = 0.60).

The 5-year survival showed that there was no significant difference in the prognosis between cSqCC and pSqCC (*P* = 0.60), and Kaplan Meier curve did not show separation between the two groups either (Figure 3).

## 4. Discussion

We have examined the differences of clinicopathologic features between central and peripheral SqCCs of the lung. Mizushima et al. reported the examination in prognostic significance between cSqCC and pSqCC [6]. Their result was similar to ours, showing no significant difference between the two groups. As for the histological pattern, Mizushima and Yousem claimed that predominance of alveolar filling pattern was found in pSqCC; on the other hand, infiltrating pattern predominated in cSqCC [5, 6]. Our examination also showed similar trend; however, probably due to small sampling size of this cohort, the data did not reach to statistical significance (*P* = 0.16). Instead, entrapped pneumocytes, detected by CK7, showed significant predominance in pSqCC (*P* = 0.04). This phenomenon might be affected by the surrounding tissue, caused by the tumor location; that is, the pSqCC is surrounded by the alveolar free air space, whereas the cSqCC is surrounded by the connective tissue of the bronchus. 

These results indicate that there may be little biological difference between cSqCC and pSqCC yet show slight difference in manner of proliferation. Considering the similarity between the two groups, it may be reasonable to apply the similar therapeutic protocol to the patients of both cSqCC and pSqCC. There were some reports suggesting similarity between pSqCC and adenocarcinoma morphologically. Although our study did not compare the histological findings of SqCC and adenocarcinoma, no SqCC reacted with Napsin A, and only a few cases of SqCC showed weak reaction for TTF-1. The presence of emphysema in pSqCC is an interesting finding. Considering the description by the recent reports that combined emphysema and fibrosis (CPFE) has higher chance of cancer occurrence [7], there may be a biological association between emphysema with or without fibrosis and carcinogenesis. Kawasaki et al. reported interesting findings of the alteration of p53 gene in the atypical squamous metaplasia in the patients with idiopathic pulmonary fibrosis [8]. Such alteration of tumor suppressor genes including p53 may be induced by the smoking-related injury causing emphysema and/or fibrosis. Increase of residual volume due to emphysema may also increase the risk of cancer due to its poor clearance of carcinogenic agents. Mizushima et al. made a prognostic comparison between pSqCC and cSqCC and concluded that the location of the tumor may not have significant influence on the prognosis [6]. 

Our result also showed no prognostic difference in pSqCC and cSqCC. One of the limitations of our study is the small number of cases. To confirm our conclusions, larger numbers of cases from different cohorts are necessary. Another limitation is that this is a retrospective research and the materials were limited to the patients who underwent surgical resection. Therefore, selection bias may exist. A part of the reason why the ratio of peripheral SqCC is high in our cohort is due to this selection bias. Higher numbers of central SqCC cases may not have undergone surgery due to its direct invasive manner to adjacent organs. 

In summary, central and peripheral SqCCs may not be biologically and prognostically different except in ways of proliferation. In addition, emphysema and fibrosis may increase the risk of SqCC occurrence. 

## Figures and Tables

**Table 1:** Patient characteristics of central and peripheral squamous cell carcinomas (*n* = 50).

	Central (*n* = 15)	Peripheral (*n* = 35)	*P* value
Age			
Median	72	75	
Range	58–88	62–86	0.21
Sex			
Male	14	34	
Female	1	1	0.53
Smoking			
BI index median	960	1000	
BI index range	400–1880	0–2000	0.66
Stage I	7	20	0.37
Stage II	5	10	0.83
Stage III	3	3	0.29
Stage IV	0	0	
Severe emphysema (HRCT)	2	17	0.02
Interstitial pneumonia (HRCT)	5	21	0.08

**Table 2:** Histological findings of central and peripheral squamous cell carcinomas.

	Central	Peripheral	*P* value
	Positive cases/total cases (%)	
Alveolar filling pattern	1/12 (8)	8/30 (27)	0.16
Infiltrating pattern	11/12 (92)	22/30 (73)	0.16
Inflammation	9/12 (75)	25/30 (83)	0.85
Necrosis	12/12 (100)	27/30 (90)	0.35
Fibrosis inside the tumor	12/12 (100)	28/30 (93)	0.51
Keratinization	8/12 (67)	19/30 (63)	0.57
Capsule	3/12 (25)	4/30 (13)	0.91
Background lung fibrosis	4/11 (36)	14/30 (47)	0.41
Traction bronchiectasis	6/11 (55)	9/30 (30)	0.14
Visceral pleural invasion	7/15 (47)	19/35 (54)	0.62
Emphysema (microscopic)	6/13 (46)	24/31 (77)	0.039
Interstitial pneumonia (microscopic)	2/13 (15)	17/31 (55)	0.015

**Table 3:** Immunohistochemical findings of central and peripheral squamous cell carcinomas.

	Central	Peripheral	*P* value
	Positive cases/total cases (%)	
CK7	5/10 (50)	16/30 (53)	0.85
p63	9/9 (100)	22/27 (81)	0.16
CK14	6/9 (67)	13/27 (48)	0.34
TTF-1	1/10 (19)	2/28 (7)	0.77
Napsin A	0/10 (0)	0/28 (0)	N/A
CK34*β*E12	10/10 (100)	27/30 (90)	0.3
CK5/6	3/10 (30)	11/29 (38)	0.65
p53	8/10 (80)	21/28 (75)	0.56
Entrapped pneumocytes (CK7)	1/10 (10)	14/30 (47)	0.04
